# Desert Sand in Alkali-Activated Fly Ash–Slag Mortar: Fluidity, Mechanical Properties, and Microstructure

**DOI:** 10.3390/ma18143410

**Published:** 2025-07-21

**Authors:** Wei Wang, Di Li, Duotian Xia, Ruilin Chen, Jianjun Cheng

**Affiliations:** College of Water Conservancy & Architectural Engineering, Shihezi University, Shihezi 832003, China; dw210601@163.com (W.W.); lidi2543@foxmail.com (D.L.); strive_crl@163.com (R.C.); chengjianjun@shzu.edu.cn (J.C.)

**Keywords:** alkali-activated mortar, high-calcium fly ash, desert sand replacement ratio, mechanical properties, microscopic analysis

## Abstract

The role and performance of desert sand in alkali-activated mortar remain insufficiently understood. To address this knowledge gap, this study systematically investigates the fluidity, mechanical properties, and microscopic morphology of alkali-activated mortar with varying desert sand substitution rates (DSRR, 0–100%). The key findings reveal that a low DSRR (10–20%) enhances mortar fluidity and reduces drying shrinkage, though at the cost of reduced compressive strength. At 40% DSRR, the mortar exhibits elevated porosity (12.3%) and diminished compressive strength (63 MPa). Notably, complete substitution (100% DSRR) yields a well-structured matrix with optimized pore distribution, characterized by abundant gel micropores, and achieves a compressive strength of 76 MPa. These results demonstrate that desert sand can fully replace river sand in alkali-activated mortar formulations without compromising performance. Microstructural analysis confirms that desert sand actively participates in the alkali activation process. Specifically, the increased Ca^2+^ content facilitates the transformation of amorphous gels into crystalline phases. It also found that desert sand could make the fly ash more soluble, affecting the alkali activation reaction.

## 1. Introduction

Concrete is a composite construction material composed of cementitious binder (typically Portland cement), mineral aggregates (including fine aggregates like sand and coarse aggregates such as gravel), water, and optional chemical admixtures [1]. It is a composite construction material that offers significant advantages over traditional building materials like metal and wood, primarily due to the global availability and cost-effectiveness of its raw materials. However, this material system presents notable environmental challenges, particularly the ecological damage associated with cement production processes [1]. Cement is a commonly used material, yet its “two grinds and a burn” preparation process releases significant amounts of CO_2_ [2]. According to statistics, CO_2_ from cement production will account for 16–24% of anthropogenic carbon emissions by 2050 [3]. The demand for Portland cement alternatives in the construction industry is stronger than ever [4]. In recent years, alkali-activated materials (AAMs) have garnered significant attention in the research community. AAMs are a class of inorganic non-metallic materials formed through the chemical reaction of aluminosilicate precursors under alkaline conditions [5,6]. They are considered a type of green, low-carbon cementitious material with high development potential. AAMs have lower CO_2_ emissions compared to cement [7]. They also have excellent properties, including high strength [8,9], high-temperature resistance [10,11], and corrosion resistance [12,13]. As a result, they have become a popular research topic both domestically and internationally. After researching extensively, the methods for preparing various alkali excitation systems are becoming increasingly perfect [14,15,16,17,18,19,20,21,22,23]. Scholars have mainly used industrial by-products rich in silica and aluminate as precursor materials. These materials are activated using a combined alkali solution of Na_2_SiO_3_ and NaOH to prepare AAMs [24,25,26]. There are more studies using fly ash (FA) as a precursor material [27,28], especially low-calcium FA (CaO < 10%) because of its high emissions and relatively stable chemical composition [29]. In some regions, emissions of high-calcium FA (HCFA) have exceeded those of low-calcium FA [30]. However, there are few recent studies on the alkali activation of HCFAs [31,32]. CaO ≥ 10% HCFA vitreous material has a high content of free CaO, which affects its performance. On the one hand, it can significantly improve its pozzolana activity, making it cemented with high-grade low-calcium fly ash [33,34,35], which is an advantage as a precursor material. On the other hand, too much free CaO produces additional gels in AAMs [36], which prevents gel formation and is not conducive to the development of strength. Furthermore, FA-based AAMs typically require high-temperature curing to develop early mechanical strength [37,38], which undoubtedly increases cost, energy consumption, and application difficulty. To address these issues, scholars have experimented with the use of HCFA in combination with cement and have found that the early strength of AAMs can be improved [39]. However, the use of cement increases CO_2_ emissions, making it a less popular option. Previous studies have shown that mixing HCFA with finely ground granulated blast furnace slag (GGBS) results in a denser gel, which improves the early strength of AAMs [40,41].

In addition to the cementitious material, the composite material also includes a large amount of aggregate, more than 70% [42]. River sand is the most commonly used aggregate, and it is widely used in cement-based materials and alkali-activated materials. However, overexploitation of river sand is disrupting the ecological balance, and finding new sources of sand has become a global challenge [43]. Desert sand may reduce transportation energy consumption under specific geographical conditions (e.g., near desert areas), but its comprehensive environmental benefits need to be further verified in combination with LCA. It has been found that the particle size of desert sand is very fine, making it difficult to meet the gradation criteria and not suitable for use as a fine aggregate alone [44,45]. The mechanical properties of composites depend on the optimal filling of the particle components [46]. Studies have shown that DSRR significantly affects the performance of concrete [47,48]. Better results can be obtained by mixing desert sand with well-graded river sand [49]. It has been shown that it is feasible to use desert sand to prepare high-performance concrete [50,51,52]. Zhang [53] formulated desert sand high-performance concrete according to the dense accumulation theory, and the test results showed that the concrete had good fluidity, mechanical strength and shrinkage, and met the standard requirements of high-performance concrete. In summary, desert sand can be used as a fine aggregate in cementitious materials and even in the production of desert sand high-performance concrete. However, there are few research results on the application of desert sand in the Loess Plateau. Some researchers have applied desert sand to AAM and explored its mechanical properties [54,55,56,57]. Unfortunately, many studies only treat desert sand as an aggregate, ignoring the potential activity of reactive oxides (SiO_2_) and amphoteric oxides (Al_2_O_3_) in desert sands. In addition, scholars have found that desert sand can only partially replace river sand in AAMs [57,58]. Fortunately, 100% DSRR was achieved in this study, and the mortar showed excellent mechanical properties. However, there are still some areas for improvement that hopefully will be addressed in future research.

In the context of the development of the global low-carbon economy and the shortage of river sand resources, it is of great significance to continue the research on AAM, especially HCF-AAM. Another important study is that desert sand has great potential to completely replace river sand as AAM fine aggregates, which has a positive effect on expanding the utilization of fly ash and mineral fines. Therefore, the fluidity and mechanical properties of alkali-activated mortar under different DSRRs were studied. At the same time, scanning electron microscopy was used to observe the microscopic morphology changes in alkali-activated mortar. The results of this paper will provide a theoretical basis for the application of desert sand in AAM.

## 2. Experimental Program

### 2.1. Raw Materials

**Figure 1:**
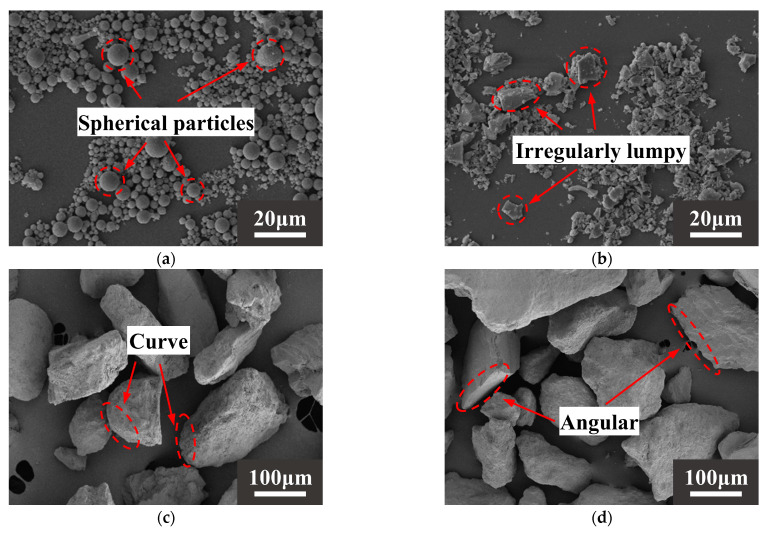
SEM images of (**a**) HCFA, (**b**) GGBS, (**c**) DS, (**d**) RS.

**Figure 2:**
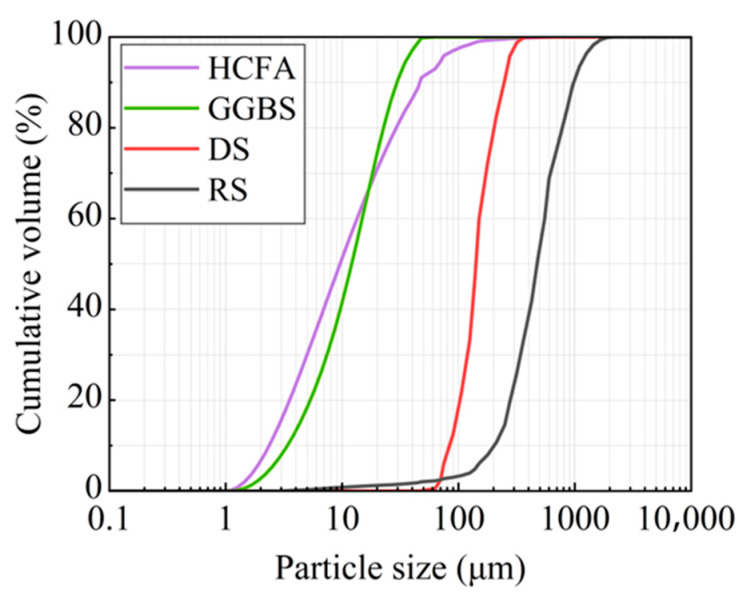
Particle size distribution of raw materials.

**Figure 3:**
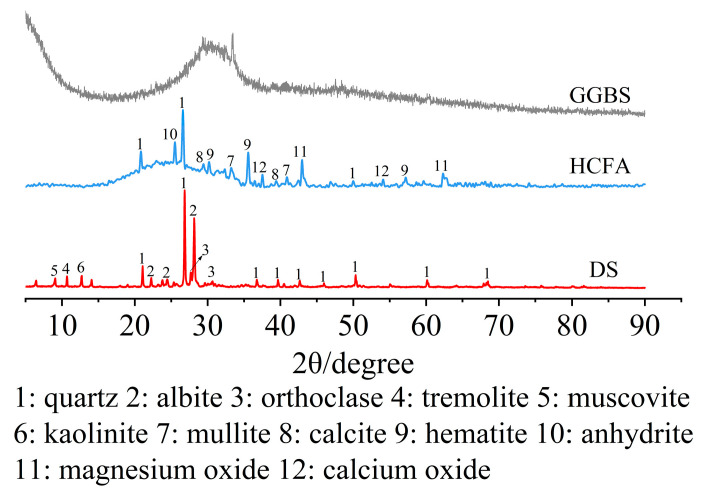
XRD patterns of DS, HCFA and GGBS.

The raw materials came from Xinjiang, China. The alkali activation precursor materials are HCFA (C grade) and GGBS (S95 grade). The aggregates are desert sand (DS) and river sand (RS). Table 1 shows the physical properties of the two types of sand. The chemical composition of the raw materials is shown in Table 2. Figure 1 shows an SEM image of a solid material. FA is spherical particles of different sizes, and GGBS is mostly irregular lumps. DS has a looser surface with rounded textured edges, while RS has a flat surface with angular textured edges. The river sand was passed through a 1.18 mm square hole sieve to reduce aggregate particle size differences. The particle size distribution of the material showed that the median particle size of DS (50% cumulative volume) was 143.18 μm, containing many very fine particles (VFPs < 175 μm), as shown in Figure 2. DS has a high CaO content. Figure 3 shows the XRD results for DS, HCFA, and GGBS.

### 2.2. Alkali Solution

The modulus (M = n(SiO_2_)/n(Na_2_O)) of the undisturbed sodium silicate solution used in the test was 3.28, the contents of Na_2_O and SiO_2_ were 8.35% and 26.54%, the water content was 65.11%, and the Baumé degree was 39.3° Bé. Flake sodium hydroxide (purity ≥ 96%) was added to the undisturbed sodium silicate solution to prepare an alkali activation solution with a modulus of 1.3. We used a glass rod to quickly stir the solution until there was no visible precipitate in the mixed solution. Finally, we left the membrane-covered mixed solution for 24 h. The water came from the laboratory.

### 2.3. Sample Preparation

On the basis of our previous research [59], we further explored the effects of DSRRs (0%, 10%, 20%, 30%, 40%, 50%, 60%, 80%, and 100%) on the properties of alkali-activated mortar. W/B remained constant at 0.35 during the experiment (the water in the water-to-binder ratio was the sum of water in the sodium silicate solution and the additional water). Table 3 shows the specific experimental mixing ratio and sample number. DS-10% represents replacing river sand with 10% desert sand. Other numbering methods are the same.

**Figure 4:**
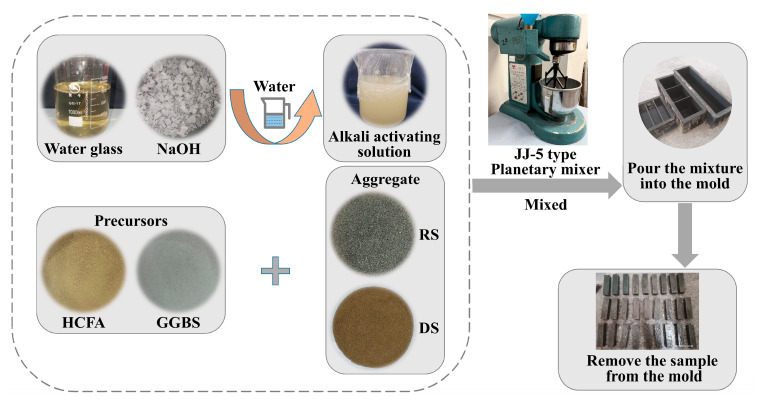
HCFA-based alkali-activated mortar preparation process.

First of all, the solid material was poured into a cement sand planetary mixer, the solid powder was stirred to a homogeneous level, and the alkali solution and water were added. The mixture was poured into a mold and shaken for 60 s to remove air bubbles. Finally, we then covered it with a film and placed it in a curing oven (20 ± 1 °C, RH ≥ 95%). After 24 h of curing, the sample was demolded and labeled, and placed again in a standard curing chamber to cure for the time required for the experiment. Figure 4 shows the preparation process.

### 2.4. Methods

#### 2.4.1. Fluidity Test

The fluidity test was based on GB/T 2419–2005 (Chinese Standard) [60]. First, the mixture was placed in a truncated conical mold. It was first compacted, then lifted vertically. When the truncated cone mold was separated from the mixture, the flow tester was activated with 25 beats at a rate of once per second. Finally, two mutually perpendicular directions were selected to measure the diffusion diameter. Two averages were taken as the fluidity result.

#### 2.4.2. Mechanical Properties Test

**Figure 5:**
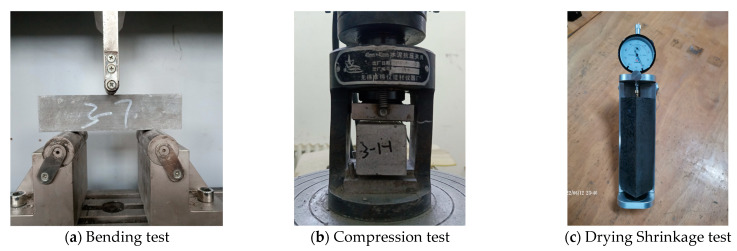
Test methods.

For the mechanical properties test, we referred to GB/T 17671-2021 (Chinese Standard) [61]. Flexural strength was measured using the three-point bending method and a 40 mm × 40 mm × 160 mm prism, as shown in Figure 5a. After the flexural test, a compressive strength test was performed using 6 solidified blocks with a loading area of 40 mm × 40 mm, as shown in Figure 5b. Finally, the cubic average and the sixth-time average were taken as the results of the mechanical properties.

#### 2.4.3. Drying Shrinkage Test

For the drying shrinkage test, we referred to JGJ/T 70-2009 (Chinese standard) [62] and used 40 mm × 40 mm × 160 mm prisms with copper nails, as shown in Figure 5c. After the initial length was determined, the mortar samples were placed in a chamber (20 ± 2 °C, RH: 60 ± 5%) and then tested for 7 d, 14 d, 21 d, 28 d, 42 d, and 56 d. Finally, the average of the three times was taken as the dry shrinkage result.

#### 2.4.4. Elastic Modulus Test

For the modulus of elasticity test, we referred to JGJ/T 70-2009 [62], using 70.7 mm× 70.7 mm× 229 mm prisms. First, we measured the size of the sample (accurate to 1 mm). If the difference between the actual size and the design size does not exceed 1 mm, it can be calculated based on the design size. The samples were tested using the loading procedure specified in JGJ/T 70-2009. Finally, the elastic modulus of the mortar was obtained by averaging the values obtained from the three samples. Three averages were taken as the result of the modulus of elasticity.

#### 2.4.5. Microstructure Characterization

First, the samples were soaked in absolute ethanol for 24 h. Then, they were dried in an oven at 60 °C for 48 h. Finally, we cut the sample into small pieces.

XRD patterns were obtained using the Bruker AXS-D8 model (5–90°, 5°/min) (Karlsruhe, Germany). SEM was tested with the ZEISS Sigma 300 model (Oberkochen, Germany). In addition, a Mack AutoPore V 9600 instrument (0.23–33,000 psi, 790 μm–5 nm) was used for mercury-induced porosity (MIP) testing (Micromeritics Instrument Corp., Norcross, GA, USA). Thermogravimetric and differential scanning calorimetry was carried out using an SDT 650 synchronous thermal analyzer (TA Instruments, New Castle, DE, USA).

## 3. Results and Discussion

The test results of the fluidity, compressive strength, flexural strength, and elastic modulus of the mortar under different DSRRs are shown in Table 4.

### 3.1. Fluidity

**Figure 6:**
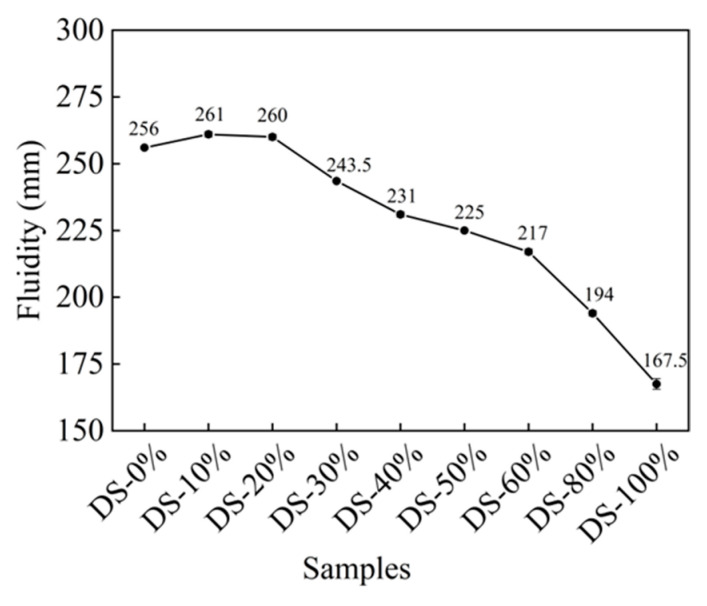
Relation between fluidity and desert sand replacement ratio.

Figure 6 shows the relationship between fluidity and DSRR. When the DSRR was 0%, the fluidity was 256 mm, which is good. After this, there was a slight increase in fluidity as the DSRR increased. The fluidity of the mortar was at the maximum when the DSRR was 10%—261 mm—which is 2% higher than when the DSRR was 0%. When the content was more than 20%, the fluidity of the mortar continued to decrease with the increase in the content. The fluidity was the smallest when the content was 100%, and the specific content was 34.57 when the content is 0%.

When the solid–liquid ratio was 10~20%, the fluidity was improved. The surface of the DS was smooth (see Figure 1). When it is mixed with river sand, this can create a “ball” effect [63]. In addition, DS can fill pores and release the water and slurry that fill those pores [64,65], meaning the friction between the slurries is reduced and the fluidity is improved. However, DS has a high specific surface area (see Table 1) and high water absorption capacity. In the case of fixed W/B, the increase in DSRR leads to a thinning of the slurry around the aggregate, increasing the friction between the two and decreasing fluidity [66].

### 3.2. Mechanical Properties

#### 3.2.1. Compressive Strength

**Figure 7:**
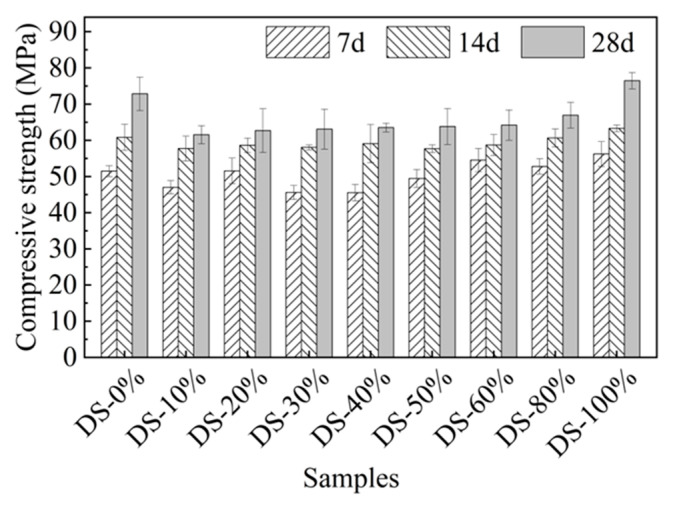
Relation between compressive strength and desert sand replacement ratio.

Figure 7 shows the relationship between compressive strength and DSRR. When the content was 10%, the compressive strength was 61.52 MPa, which is 15.54% lower than that when the content was 0%. When the DSRR was 10~80%, the intensity grew slowly. When the DSRR was increased to 100%, the compressive strength reached its maximum value (76.45 MPa). Compared to the samples with DSRRs of 0% and 80%, they improved by 4.96% and 14.28%, respectively. At 7 d, the compressive strength of some samples with a high DSRR was lower than that of samples with a low DSRR. With the increase in age, the 14-day compressive strength of high-content and low-content mortar was similar, and the 28-day compressive strength was also similar. These results suggest that DSRR may affect the early alkali activation response rate of alkali-activated mortars. Table 5 shows the differences in the compressive strength of alkali-activated materials in this paper and other studies.

As the DSRR increases, the strength of the material decreases first and then slowly increases, unlike that shown in the study, which first increased and then decreased [67]. This may be due to the following reasons. On the one hand, DS is very small and rounded with edges. The accumulation of DS on the surface of river sand increases the slippage between aggregates and reduces the strength of the sample [66]. In addition, it is also associated with VFPs in the desert. It has been shown that VFPs in desert sands have heterogeneous nucleation and volcanic ash effects in alkaline environments [68,69]. As the DSRR increases, the number of VFPs increases. These VFPs can fill the pores between the slurries and reduce the porosity [70], which is beneficial for improving strength. In addition, desert sand contains a large amount of silicon–aluminum substances (see Table 2), which can increase the concentration of silicon–aluminum substances to a certain extent. The alkali activation generates more active gels, which improves the bonding properties between the slurry and the aggregate, making the matrix denser, which was reflected in the microscopic analysis. Figure 7 also shows that the average intensity of a sample with a DSRR of 10–80% is about 13% lower than that of a sample with a DSRR of 0%. When the DSRR is 100%, the compressive strength of the specimen is the highest. If only the compressive strength is considered, it is feasible for DS to completely replace RS in the preparation of alkali-activated mortar, but the possibility that desert sand is affected by the terrain cannot be ruled out.

In this paper, the DSRR = 100% sample had the highest compressive strength of 76.45 MPa and a porosity of 6.8%. In the mix ratio of this paper, the mineral powder content was 40%, so the early strength of the sample developed rapidly, and the compressive strength was able to reach about 50 MPa in 7 days, which effectively makes up for the shortcomings of the early strength of the fly ash-based alkali-activated material. The strength in the later stage was mainly provided by fly ash; the growth rate of compressive strength in 14 days and 28 days ranged from 7.59~29.75% and 6.57~19.78%, respectively, and the strength improvement rate slowed down. The compressive strength and porosity of the DSRR = 100% samples in this paper are better than those in other studies, which is attributed to the combination of slag powder and high-calcium fly ash forming a relatively dense gel product and the fact that desert sand plays a role in promoting the formation of a gel during alkali activation, resulting in a high compressive strength and low porosity for the sample.

#### 3.2.2. Flexural Strength

**Figure 8:**
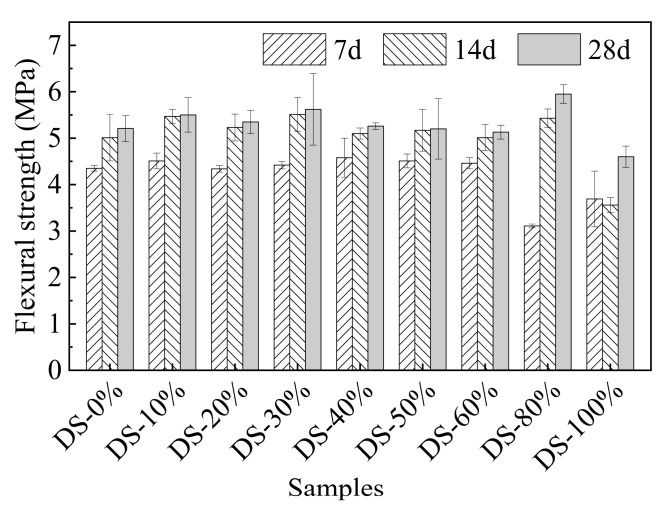
Relation between flexural strength and desert sand replacement ratio.

Figure 8 shows the relationship between flexural strength and DSRR. When the DSRR changes in the range of 0%~60%, there is no significant difference in flexural strength (*p*-value is 0.132). When the DSRR is 80%, the flexural strength reaches the maximum value (5.95 MPa), which is 14.24% higher than that when the DSRR is 0%. When the DSRR continues to increase to 100%, the minimum flexural strength (4.60 MPa) occurs, which is 11.76% lower than that when the DSRR is 0%.

On the one hand, in a highly alkaline environment, VFPs in desert sand are active and participate in some reactions, promoting gel formation and improving the compactness of the mortar [71]. On the other hand, too high a DSRR will thin the slurry surrounding the aggregate [72], resulting in a weakening of the slurry–aggregate interface, which ultimately affects the flexural strength of the material. The results show that the flexural strength of desert sand is between 0%~60%, and there is little difference in flexural strength between the two because the gain effect of VFPs compensates for the adverse effects of desert sand. When the DSRR is 80%, the positive effects of VFPs outweigh the negative effects, so the flexural strength increases. However, when the DSRR is 100%, the negative effects outweigh the positive effects, and the flexural strength decreases.

### 3.3. Drying Shrinkage

**Figure 9:**
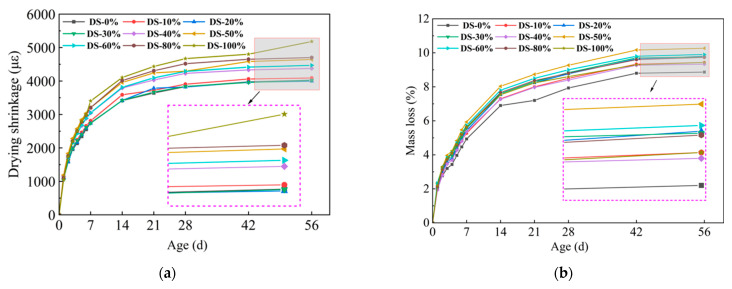
Relation between (**a**) drying shrinkage, (**b**) mass loss, and desert sand replacement ratio.

Figure 9a shows the relationship between drying shrinkage and DSRR. Drying shrinkage developed rapidly in the early stage and gradually stabilized with the increase in age. At 56 days, the sample with a dry shrinkage rate of 20% had the lowest dry shrinkage rate (3997.08 με), and there was no significant difference compared with the sample with a dry shrinkage rate of 0%. The maximum shrinkage value (5179.91 με) occurred when the shrinkage rate was 100%, which was 28.7% higher than that when the shrinkage rate was 0%, and the change range was larger. The test results show that the dry shrinkage performance of alkali-activated mortar is greatly affected by DSRR.

**Figure 10:**
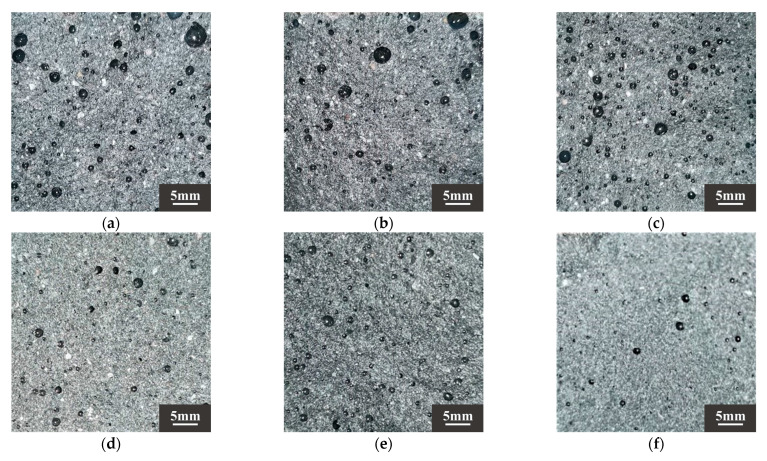
Sample sections of (**a**) DS-0%, (**b**) DS-20%, (**c**) DS-40%, (**d**) DS-60%, (**e**) DS-80%, (**f**) DS-100%.

According to Ref. [70], drying shrinkage depends on the fineness of the aggregate. DS can play a filling role, optimize the pore structure of the substrate, and make the substrate more dense. The results showed that when the DSRR was 10–30%, the sample exhibited a small drying shrinkage rate. Studies have shown that the composition of aggregates affects the movement of water between slurries [73]. The particle size of DS is smaller, and as the DSRR increases, the ability of the fine aggregate to limit moisture within the matrix decreases, resulting in high drying shrinkage rate for the mortar. In addition, the use of larger aggregate particles, such as river sand, will create more macropores in the matrix. When the same amount of water evaporates, the water in the material tends to remain in the macropores, resulting in lower pore pressure and reduced drying shrinkage of the material [74]. Smaller aggregate sizes, such as desert sand, promote the formation of capillary pores in the matrix [65]. As the reaction progresses, these capillaries are further converted into capillary micropores (see Figure 10), which causes higher shrinkage stresses and results in a higher drying shrinkage rate for the sample.

Figure 9b shows the relationship between the mass loss rate and the DSRR. It has been shown that there is a correlation between drying shrinkage and mass loss in alkali-activated materials [75]. Water evaporation occurs during microstructure formation and pore refinement within the matrix [76]. When the dry shrinkage ratio is 100%, the dry shrinkage of the mortar is the largest, but because of its good refinement effect on the hole, the strength is higher. This is also shown in the hole structure analysis below. In addition, the alkali activation reaction requires the early consumption of water in the matrix. When the reaction progresses to a later stage, the mass loss of the sample is flattened due to the free water produced by the polycondensation reaction [77].

### 3.4. Elastic Modulus

**Figure 11:**
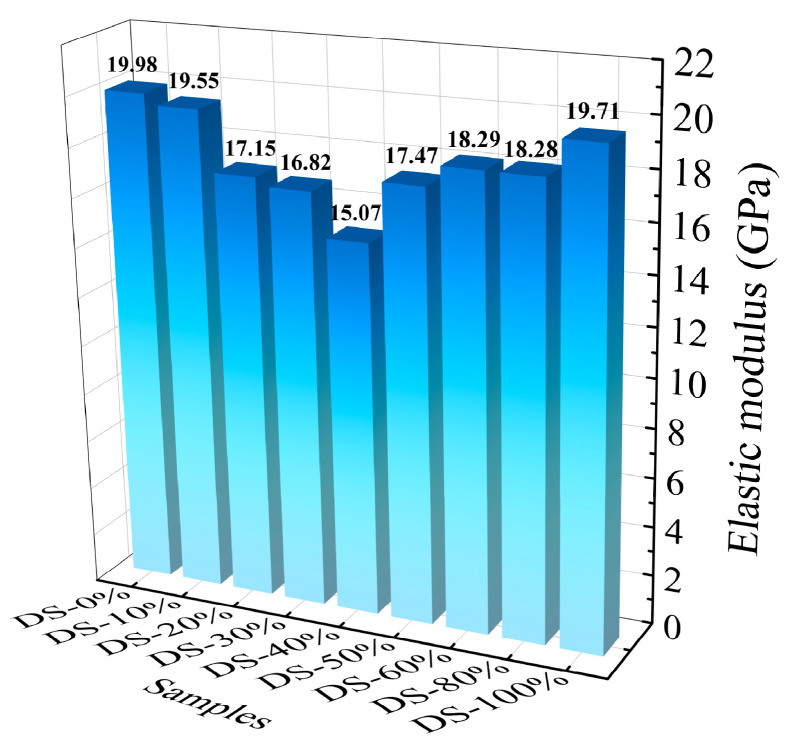
Relation between elastic modulus and desert sand replacement ratio.

Figure 11 shows the relationship between the modulus of elasticity and the DSRR. When the DSRR is 0%~40%, the elastic modulus of the mortar decreases from 19.98 GPa to 15.07 GPa. After this, the modulus of elasticity increases. When the DSRR is 100%, its modulus of elasticity is 19.71 GPa. The elastic modulus of the mortar decreases first and then increases, similar to the change in compressive strength [78]. Combined with the mechanical property test, the improvement in DSRR has positive and negative effects on the mechanical properties of the mortar, which in turn affects the elastic modulus of the mortar. In addition, a larger DSRR also increases the number of VFPs, improving the overall performance of the mortar and, thus, the sample performance.

### 3.5. Microscopic Analysis

#### 3.5.1. XRD

**Figure 12:**
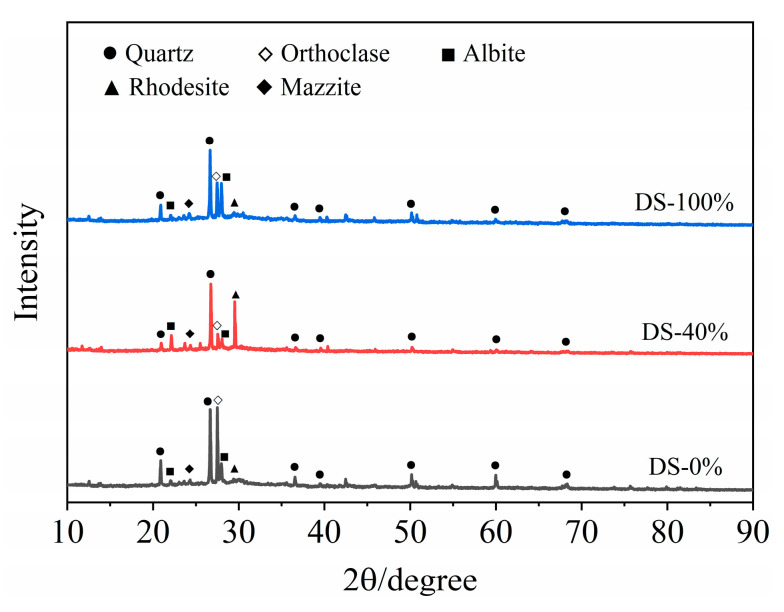
XRD of samples at 28 days.

The XRD test results of the mortar at 28 days of age are shown in Figure 12. Sharp peaks appear in the range of 2θ in the range of 20°~40°, indicating that the crystallinity of the hydration products was good. The results showed that the hydration products of the samples were basically the same under different DSRR conditions, mainly composed of quartz, orthoclase, albite, rhododite, rhododochrolite and needle zeolite, but their contents changed greatly. The diffraction peak of quartz decreases with the increase in DSRR. On the one hand, this is the reaction of the active SiO_2_ in an alkaline environment. On the other hand, desert sand can promote the dissolution of HCFA and affect the alkali activation reaction. In addition, desert sand contains a large amount of SiO_2_ (see Table 2). The decrease in the intensity of the diffraction peak can also indicate that desert sand dissolves under alkali activation and participates in part of the alkali activation reaction. Therefore, the strength of the sample will still develop at a later stage, and the mortar has a high mechanical strength. As can be seen in Figure 12, there is a significant spike when the DSRR is 40%; it is the diffraction peak of the rhodochrosite phase, located at 2θ = 29.5°. When the DSRR is 100%, the intensity of the diffraction peak of the product decreases. According to the analysis in Table 2, it can be seen that desert sand contains a large amount of CaO. Ca^2+^ has been described as a substitute for Na^+^ in gels [79]. Due to the increase in DSRR, more desert sand is involved in the reaction. Ca^2+^ facilitates the conversion of amorphous N-A-S-H gels to C-A-S-H gels with crystallinity [80]. There are more and more C-A-S-H gels in the matrix, and the structure is becoming denser and denser. In addition, DS and FA have similar chemical compositions (see Table 2), which reinforces the idea that desert sand will be involved in partial alkali activation reactions. The diffraction peaks of orthoclase and albite at 2θ = 27.5° and 28° change from weak to strong with the increase in DSRR. Orthoclase and albite are mineral phases in desert sands (see Figure 3). On the one hand, the dissolution of VFPs in an alkaline environment is limited. On the other hand, the compressive strength exhibits a slow rise due to the increase in C-A-S-H gel, which hinders the dissolution of silicon–aluminum substances.

#### 3.5.2. SEM

**Figure 13:**
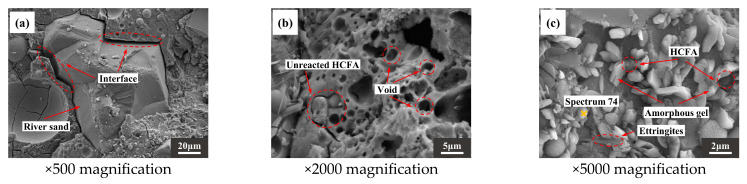

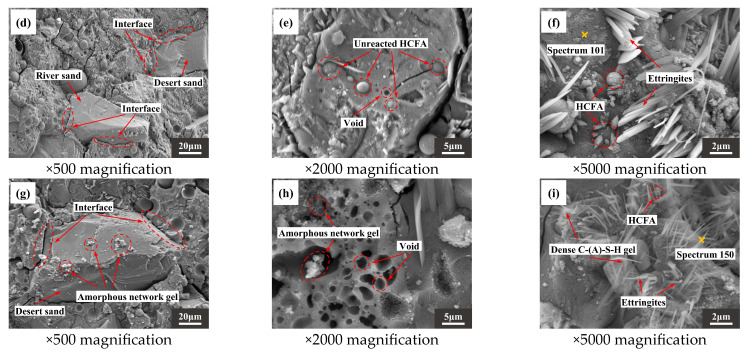
SEM images of the following (**a**–**c**): DS-0%, (**d**–**f**): DS-40%, (**g**–**i**): DS-100%.

Figure 13a,d,g are photomicrographs of aggregate and slurry bonding. It can be seen that there is a clear dividing line where the slurry meets the RS, with a wide crack between the two. However, the slurry is more tightly bound to the DS. In addition, when the DSRR is 100%, it can be observed that there is gel attached to the surface of the desert sand in Figure 13g. The interface between the matrix and the aggregate is enhanced, and the mechanical strength is improved. When the DSRR is 0% and 40%, there are undissolved fly ash particles, as shown in Figure 13b,e. When the DSRR reaches 100%, it is difficult to see the exposed fly ash particles, suggesting that the DSRR affects the degree of reaction of alkali activation. Interestingly, Figure 13h shows some gel-filled voids, thereby refining the pore structure within the matrix. Figure 13c,f,i show varying degrees of morphology of hydrate products in the sample matrix for different DSRRs. When the DSRR is 0%, there are granular hydration products in the fly ash that are encased in the fly ash and form aluminite around it, and when the DSRR increases to 40%, the amount of aluminite in the matrix increases. When the DSRR reaches 100%, the aluminite in the hydration product is significantly reduced and is replaced by dense gel hydration products and needle-like crystals, and the specimen has a higher strength.

**Figure 14:**
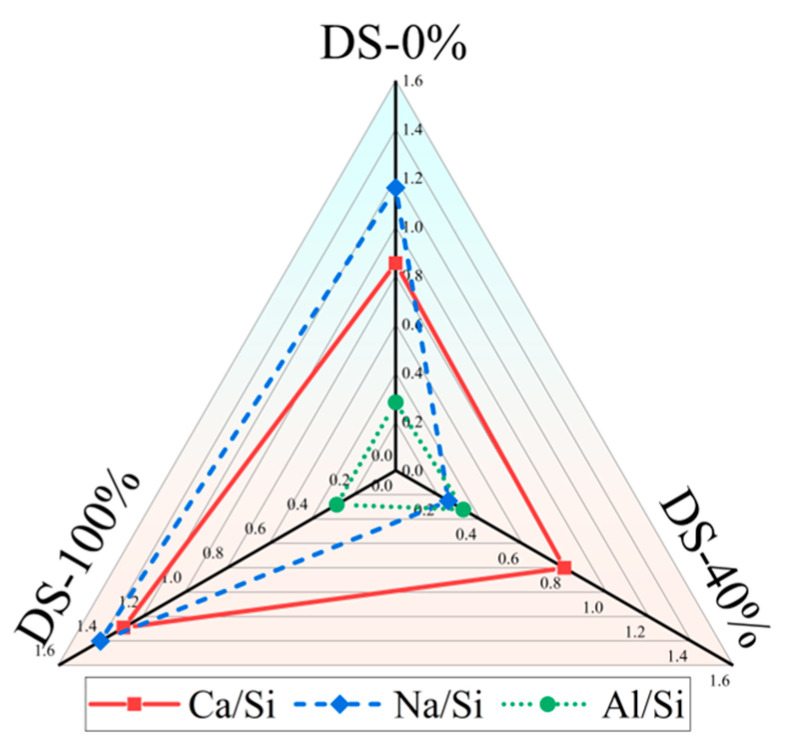
Changes in the elemental ratio at different desert sand replacement ratios.

There is a significant positive correlation between Ca/Si and compressive strength [81,82]. Table 6 shows the EDS test results for the samples. Figure 14 shows that Ca/Si tends to decrease and then increase as DSRR increases. The compressive strength of the specimen also shows the same trend, which provides strong support for the macroscopic mechanical strength analysis. When the DSRR is 100%, the sample has a high Ca/Si, which may explain why the mortar can exhibit high strength when the DSRR is 100%.

#### 3.5.3. MIP

**Figure 15:**
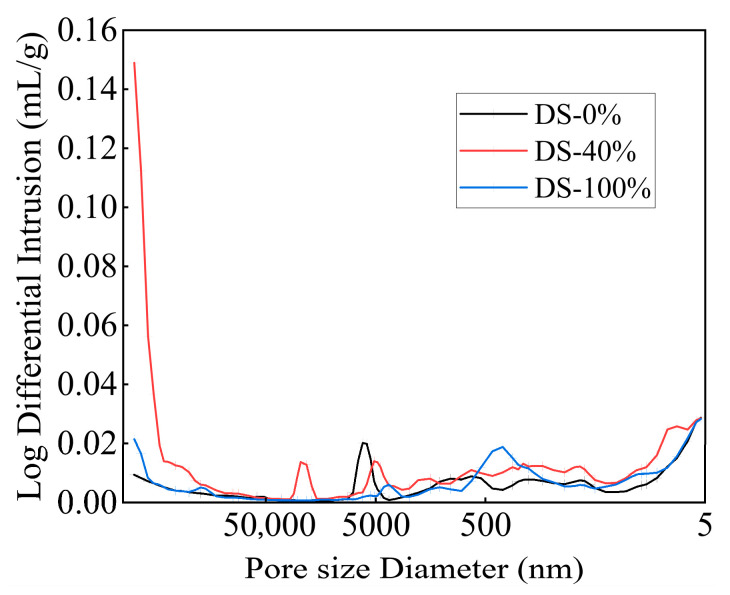
Pore diameter distribution of samples at different desert sand replacement ratios.

The pore size distribution curves of the mortar under different DSRRs are shown in Figure 15. While the pore size distribution curves of the samples have the same trend, the peak positions, peak intensities, and peak widths differ, meaning that the samples under different DSRRs had similar well types and different well sizes. When the DSRR is 0% and 100%, the distribution curve is flat, indicating that the pore size distribution is more reasonable. When the DSRR is 40%, the pore size distribution curve fluctuates obviously, the peak position shifts to the left, the pore size distribution is unreasonable, and there are many large-pore-size pores. Due to the low hydration of the sample, the gel generated in the matrix cannot fill the large pores.

**Figure 16:**
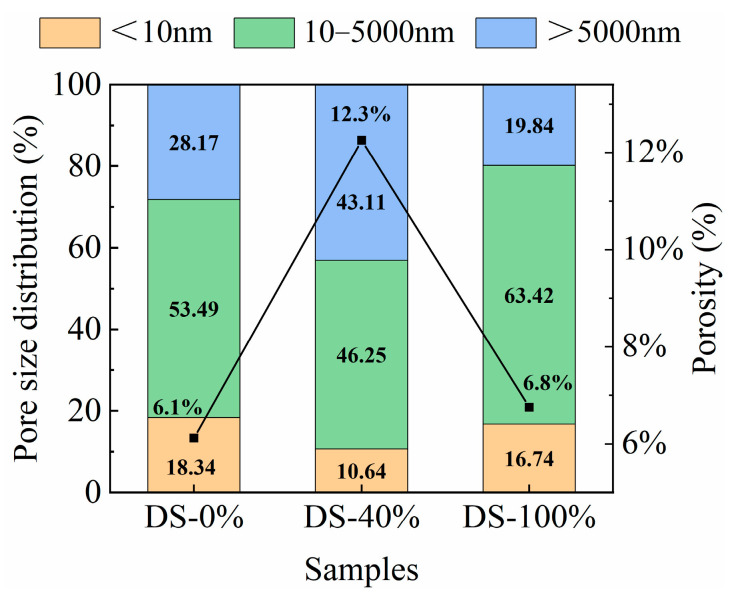
Pore size distribution and porosity of samples at different desert sand replacement ratios.

The porosity and pore type percentages under different DSRRs are shown in Figure 16. At the DSRR levels of 0%, 40% and 100%, the porosity of the sample was 6.1%, 12.3% and 6.8%, respectively, showing a trend of first increasing and then decreasing. The results showed that at 0% and 100% DSRR, the density of the matrix was better than at 40% DSRR. This explains the fact that the sample shows poor compressive strength when the DSRR is 40%, but the sample shows higher compressive strength when the DSRR is 0% and 100%. The study classified the pores into three types: gel micropores (<10 nm), capillary pores (10–5000 nm), and macropores (>5000 nm) [83]. In alkali-activated materials, a 10 nm pore is considered to be the dominant pore structure [84]. According to the calculation in Figure 16, when the DSRR is 0%, 40% and 100%, the ratios of capillary pores to gel pores in the sample matrix are 2.94, 4.35 and 3.78, respectively, so the influence of capillary pores on the drying and shrinkage performance of the sample is dominant. When the DSRR is 40%, there are fewer micropores in the specimen matrix, which also indicates that the specimen matrix is less dense and has higher strength. It was also found that there were more capillaries in the sample, which affected the drying and shrinkage properties of the sample. The data shows that the sample has the highest percentage of capillary pores when the DSRR is 100%. During the formation and development of the pore structure, these capillaries are further transformed into capillary pores, resulting in greater shrinkage stresses than when the DSRR is 0% and 40%, resulting in greater drying shrinkage of the mortar. However, an interesting phenomenon is that when the DSRR is 100%, the sample has a high compressive strength. Combined XRD and SEM analysis showed that desert sand participated in the alkali activation reaction and promoted the formation of gel under alkali activation conditions. The increase in gel micropores leads to a denser matrix, which contributes to the development of strength. The results show that the preparation of alkali-activated mortar by replacing RS with DS has positive and negative effects on the hydration, drying shrinkage and pore development of cement. The results show that when the DSRR is 100%, the specimen shows excellent strength and good pore development, which proves its feasibility.

#### 3.5.4. TG-DSC

**Figure 17:**
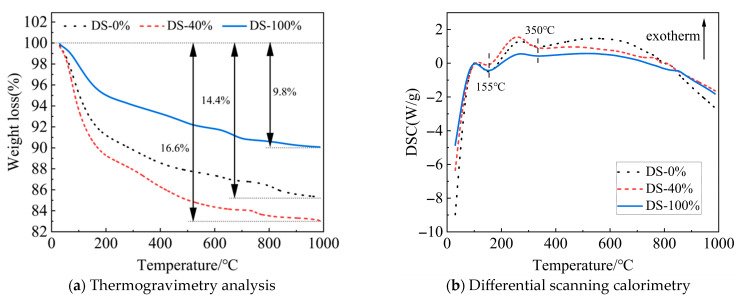
TG-DSC curves of samples at different desert sand replacement ratios.

Figure 17a shows the TG curve of the mortar. As the temperature continues to rise, the mass of the sample decreases. When the DSRR is 100%, the mass loss of the sample is small, indicating that the sample has good thermal stability at high temperatures. This may be related to well-developed internal structures and the presence of more small-diameter pores (see Figure 16). As a result, the mortar exhibits high compressive strength on a macroscopic scale. However, when the DSRR is 40%, there is a large mass loss in the sample. This reflects the poor internal structure of the matrix and its lower strength.

Figure 17b shows the DSC curve. The endothermic peaks are located around 155 °C and 350 °C, respectively. The reason for this is the loss of crystal water from C-A-S-H gel (155 °C) and sodium zeolite (350 °C) [59]. However, the XRD test results did not show the characteristic peaks of sodium zeolite. The best evidence of the low levels is the small endothermic peak area at 350 °C. The study also found that the DSRR did not affect the type of hydration product. However, the spikes are different and can be caused by different DSRRs.

### 3.6. Reaction Mechanism

**Figure 18:**
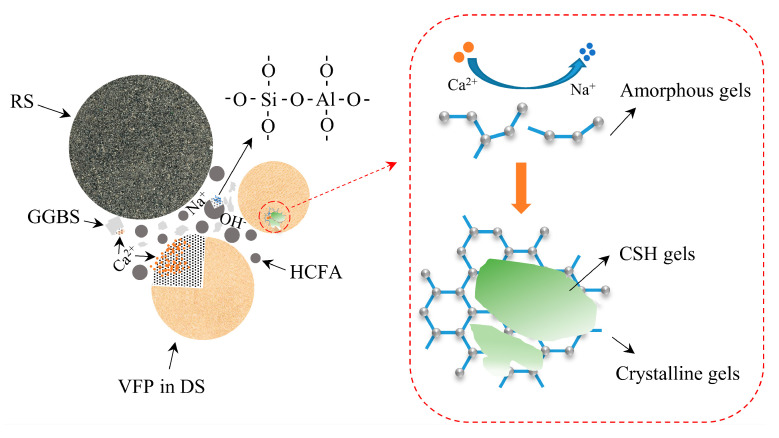
Schematic diagram of VFPs participating in alkaline activation reaction.

In this paper, a mechanistic diagram of VFPs’ involvement in the alkaline activation reaction is presented, as shown in Figure 18. Desert sand particles with a particle size of less than 175 μm have nucleation and pozzolanic effects in alkaline environments and should not be considered as a single inert component. Under alkali activation conditions, VFPs in desert sand are dissolved, which accelerates the process of the alkali activation reaction to a certain extent. As the reaction progresses, a large amount of Ca^2+^ will replace Na^+^, promoting the transition from amorphous N-A-S-H gels to crystalline C-A-S-H gels. This is the most desirable hydration product to make the substrate denser. However, the increase in C-A-S-H gels will hinder the dissolution of VFPs and the strength of the mortar will slowly rise. In addition, these gels adhere to desert sand particles, which makes the bond between the aggregate and the hydration product tighter, thereby reducing porosity and improving macroscopic mechanical properties.

## 4. Conclusions

From the results, the main conclusions we can draw are as follows:

(1) When the DSRR is 10%~20%, the fluidity and dry shrinkage performance of alkali-activated mortar can be improved. However, when the DSRR is greater than 20%, the fluidity gradually decreases, and the drying shrinkage increases. When the dry shrinkage ratio is 100%, the fluidity of the mortar is the smallest, and the dry shrinkage ratio is the largest.
(2) Compressive strength is positively correlated with the elastic modulus. When the content is 100%, the compressive strength of the mortar reaches 76 MPa. When the DSRR is between 0%~60%, the effect on the flexural strength of the mortar is not significant, and when it exceeds 60%, the effect on the flexural strength of the mortar is significant.
(3) The XRD results showed that desert sand was involved in alkali activation. With the increase in the DSRR, on the one hand, more gels were formed, which hindered the dissolution of silicon–aluminum substances. On the other hand, the amount of desert sand increased, which led to the intensification of the diffraction peaks of orthoclase and albite. Ca^2+^ can promote the transformation of amorphous N-A-S-H gels to crystalline C-A-S-H gels, thereby improving the mechanical properties of mortars.
(4) The results of scanning electron microscopy (SEM) showed that the desert sand was tightly bound to the matrix. In addition, a layer of gel was attached to the surface of the desert sand, which enhanced the interface bond between the two. The increase in DSRR accelerated the rate of the alkali activation reaction.
(5) The MIP test results show that when the DSRR is 100%, the sample has a reasonable pore size distribution. This is because more VFPs are involved in the hydration reaction, while Ca^2+^ promotes gel formation. However, there are a large number of capillaries in the matrix, which is not conducive to the drying and shrinkage of the mortar.
(6) Through this study, we can have a more comprehensive understanding of the macroscopic properties and microscopic morphology of alkali-activated mortar under different DSRRs, and it is clear that VFPs in desert sand can not only participate in alkali activation reaction but also promote gel transformation. However, at present, the specific response degree of desert sand under alkali activation is still relatively vague, and more microscopic methods are needed for in-depth research.
(7) This study can provide a theoretical basis for promoting the application of alkali activation materials in areas with abundant desert sand resources, but whether the regional characteristics of desert sand, the system composition of precursor materials, and the selection of alkali activators will have an impact on the alkali activation reaction of desert sand remains to be explored. In addition, there is no research on durability in this paper, so the variation in the material’s properties in extreme environments is not clear.

## Figures and Tables

**Table 1:** Physical properties of sand.

Sands	Bulk Density (kg/m^3^)	Apparent Density (kg/m^3^)	Fineness Modulus	Specific Surface Area (m^2^/g)	Clay Content (%)
DS	1513	2655	0.7	0.0450	2.38
RS	1596	2685	2.2	0.0095	2.47

**Table 2:** Chemical composition of raw materials.

Materials	Chemical Composition (%)										
	SiO_2_	Al_2_O_3_	CaO	Fe_2_O_3_	SO_3_	MgO	K_2_O	Na_2_O	MnO	TiO_2_	Others
HCFA	45.68	16.72	13.37	10.42	1.73	4.37	2.10	3.18	0.16	1.01	1.26
GGBS	30.92	8.82	45.73	0.49	2.56	6.96	0.60	0.96	0.62	1.64	0.72
DS	64.21	12.22	7.43	5.76	0.19	2.35	3.20	2.69	0.13	1.12	0.70
RS	69.97	12.68	2.85	4.80	0.13	1.63	3.58	3.32	0.09	0.61	0.34

**Table 3:** Mixture proportion of alkali-activated mortar.

Samples	DSRR	Na_2_O/b	Alkali Activation Solution Modulus	Component (kg/m^3^)						
				FA	GGBS	DS	RS	Na_2_SiO_3_	NaOH	Water
DS-0%	0%	6%	1.3	438.8	292.5	0	1097.0	210.6	34.6	118.9
DS-10%	10%	6%	1.3	438.8	292.5	109.7	987.3	210.6	34.6	118.9
DS-20%	20%	6%	1.3	438.8	292.5	219.4	877.6	210.6	34.6	118.9
DS-30%	30%	6%	1.3	438.8	292.5	329.1	767.9	210.6	34.6	118.9
DS-40%	40%	6%	1.3	438.8	292.5	438.8	658.2	210.6	34.6	118.9
DS-50%	50%	6%	1.3	438.8	292.5	548.5	548.5	210.6	34.6	118.9
DS-60%	60%	6%	1.3	438.8	292.5	658.2	438.8	210.6	34.6	118.9
DS-80%	80%	6%	1.3	438.8	292.5	877.6	219.4	210.6	34.6	118.9
DS-100%	100%	6%	1.3	438.8	292.5	1097.0	0	210.6	34.6	118.9

**Table 4:** The results.

Samples	Fluidity/mm	Compressive Strength/MPa			Flexural Strength/MPa			Elastic Modulus/GPa
		7d	14d	28d	7d	14d	28d	
DS-0%	256.0	51.48 (1.49)	60.81 (3.61)	72.84 (4.62)	4.35 (0.06)	5.01 (0.50)	5.21 (0.28)	19.98
DS-10%	261.0	47.00 (1.83)	57.72 (3.41)	61.52 (2.51)	4.51 (0.17)	5.47 (0.15)	5.50 (0.37)	19.55
DS-20%	260.0	51.53 (3.53)	58.60 (2.01)	62.67 (6.03)	4.34 (0.07)	5.23 (0.29)	5.35 (0.25)	17.15
DS-30%	243.5	45.59 (1.92)	58.04 (0.69)	63.08 (5.52)	4.42 (0.08)	5.51 (0.36)	5.62 (0.77)	16.82
DS-40%	231.0	45.53 (2.28)	59.07 (5.28)	63.51 (1.21)	4.58 (0.42)	5.10 (0.12)	5.26 (0.07)	15.07
DS-50%	225.0	49.45 (2.45)	57.67 (1.05)	63.80 (4.96)	4.51 (0.15)	5.17 (0.45)	5.20 (0.65)	17.47
DS-60%	217.0	54.54 (3.21)	58.68 (2.87)	64.19 (4.19)	4.46 (0.12)	5.01 (0.28)	5.13 (0.15)	18.29
DS-80%	194.0	52.77 (2.10)	60.65 (2.47)	66.90 (3.54)	3.11 (0.04)	5.43 (0.20)	5.95 (0.20)	18.28
DS-100%	167.5	56.20 (3.42)	63.30 (0.94)	76.45 (2.24)	3.69 (0.60)	3.56 (0.16)	4.60 (0.23)	19.71

Note: The figure in parentheses is the standard deviation of the corresponding parameter.

**Table 5:** Compressive strength comparison table.

Property	Specimen Size	28-Day Compressive Strength /MPa	Porosity %
this paper	40 × 40 × 40 mm^3^	76.45	6.8
[32]	ϕ2.54 cm × 2.54 cm	>30	-
[33]	50 × 50 × 50 mm^3^	>70	16.28
[37]	50 × 50 × 50 mm^3^	45.8	-
[38]	50 × 50 × 50 mm^3^	60	-
[39]	50 × 50 × 50 mm^3^	48.8	-
[40]	50 × 50 × 50 mm^3^	80.43	13.3
[41]	40 × 40 × 160 mm^3^	>40	>25

Note: “-” indicates that the data is not available in the literature.

**Table 6:** EDS test results.

Element Share		O	Na	Al	Si	Ca	Fe	Ca/Si	Na/Si	Al/Si
		wt%	wt%	wt%	wt%	wt%	wt%			
DSRR	Spectrum74	46.09	14.62	3.54	10.76	12.63	1.58	0.85	1.16	0.28
	Spectrum101	34.96	5.42	6.99	17.65	22.09	1.58	0.80	0.25	0.32
	Spectrum150	40.81	15.96	3.23	14.72	11.39	0.47	1.29	1.40	0.28

## Data Availability

The original contributions presented in this study are included in the article. Further inquiries can be directed to the corresponding author.
